# Ancient Norse Surgery

**Published:** 1870-06

**Authors:** 


***Ancient Norse Surgery.***

In the Sagas of Snorro Sturleson, we read of Thormod, a Scald, (A.D. 1030) who was wounded by an arrow which penetrated the chest. He broke off the projecting part and waited patiently, meanwhile assisting while other wounded men were attended. The doctress (or surgeoness) (who we hope was not jeered at by the masculine walkers of the wards,) said: "'Let me see thy wound and I will bind it.' Thereupon, Thormod sat down, cast off his clothes, and the girl [*sic*] saw his wounds, and examined that which was in his side, and felt that a piece of iron was in it, but could not find where the iron had gone in. In a stone pot she had stirred together leeks and other herbs, and boiled them and gave the wounded men of it to eat, by which she discovered if the wound had penetrated into the belly; for if the wound had gone so deep it would smell of leek. She brought some of this now to Thormod, and told him to eat of it. He replied, 'Take it away, I have no appetite for my broth;' then she took a large pair of tongs and tried to pull out the iron; but it sat too fast and would in no way come, and as the wound was swelled, little of it stood out to lay hold of. Now, said Thormod, 'Cut so deep in that thou canst get at the iron with the tongs, and give me the tongs and let me pull;' she did as he said. * * * Then Thormod took the tongs and pulled the iron out; but on the iron there was a hook, at which hung some morsels of flesh from the heart—some white, some red. When he saw that, he said, 'The king has fed us well. I am fat even at the heart roots;' and so saying he leant back and was dead."

In a subsequent reign, that of Magnus the Good, we are informed that after a great battle: "The King ordered the wounds of his men to be bound; but there were not so many doctors in the army as were necessary, so the King himself went round, and felt the hands of those he thought best suited for the business; and when he had thus stroked their palms he named twelve men, who, he thought, had the softest hands, and told them to bind the wounds of the people; and although none of them had ever tried it before, they all became, afterwards, the best doctors." From them, the chronicle further relates, "very many good doctors are descended." This ordeal of palmistry may be commended to the

Committee on Preliminary Qualifications of the American Medical Association.

